# Diagnostic Features of Discogenic Low Back Pain: A Narrative Review and Suggested Areas for Future Research and Clinical Practice

**DOI:** 10.1002/jsp2.70195

**Published:** 2026-05-28

**Authors:** William Roger Peters, Ashish D. Diwan, Mario Giuseppe Zotti

**Affiliations:** ^1^ Faculty of Health Sciences and Medicine Bond University Robina Queensland Australia; ^2^ Spine Service, Department of Orthopaedic Surgery St. George Hospital Campus Sydney New South Wales Australia; ^3^ St George and Sutherland Clinical School, the University of New South Wales Sydney Australia; ^4^ Royal Adelaide Hospital and Adelaide University Adelaide South Australia Australia; ^5^ Back/Neck Clinic Gold Coast Queensland Australia

**Keywords:** discogenic low back pain, discography, intervertebral disc, magnetic resonance spectroscopy

## Abstract

**Background:**

Chronic low back pain is a leading cause of global disability, with discogenic low back pain (DLBP), mechanically stimulated pain arising from the intervertebral disc, representing an important but often under‐recognized subtype. DLBP arises from chronic structural and metabolic changes within the intervertebral disc, leading to inflammation, nerve ingrowth, and nociceptive sensitization. Diagnosis remains difficult due to overlapping symptoms, non‐specific imaging, and the lack of a universally accepted reference standard. Accurate identification is critical for guiding effective management. This review aims to summarize and evaluate current and proposed diagnostic features of DLBP.

**Methods:**

A narrative literature review was conducted using PubMed, Scopus, and Web of Science, with supplementary hand searching and citation tracking. Studies evaluating clinical or investigative diagnostic features of DLBP were included, without restriction on study design. Studies involving neural compression, radiculopathy, or non‐DLBP were excluded. Evidence was thematically synthesized, with preference given to higher‐quality or larger studies where findings overlapped.

**Results:**

No clinical features have been consistently validated as predictive of DLBP. Flexion‐based postures, movements, and prolonged sitting demonstrate modest diagnostic value, while traditional features such as axial loading or Valsalva maneuvers lack supporting evidence. Imaging findings, including Modic changes, high‐intensity zones, and Pfirrmann grading, show limited specificity. Provocative discography is constrained by invasiveness, complications, and questionable reliability. Emerging metabolic imaging techniques demonstrate early potential as future reference standards. Evidence of clinical prediction models for DLBP remains limited.

**Conclusions:**

DLBP diagnosis remains constrained by non‐specific features and imperfect reference standards. Emerging metabolic imaging techniques warrant further validation, alongside development of robust diagnostic models to improve clinical decision making.

## 1 Background

Chronic low back pain remains one of the leading causes of disability in the world, with prevalence on the rise [1, 2]. A predominant subtype termed discogenic low back pain (DLBP) describes pain associated with the intervertebral disc (IVD) [3, 4, 5]. Situated between adjacent vertebrae, the IVD is composed of a collagen‐rich annulus fibrosus encircling a proteoglycan‐rich nucleus pulposus, and bounded by cartilaginous endplates. Nociceptors are normally found within the outer third of the annulus and are derived from sinuvertebral nerves, and branches of the ventral rami and gray rami communicantes [6, 7]. The IVDs are avascular and remain metabolically active through diffusion‐based nutrient exchange and waste clearance, creating a characteristically hypoxic environment, a key to the integrity of the extracellular matrix [8]. For many years, DLBP has been controversial with the pain generator disputed to be neurological compression rather than painful nociception within the disc itself [9], consequently DLBP was a poorly accepted entity. Despite this, the IVD has since been well established as a potent source of back pain [10, 11]. While early experimental work established that the IVD possesses sensory innervation and can reproduce pain when stimulated [10, 12]. Modern molecular research has described DLBP as an active process driven by a combination of structural, metabolic, inflammatory and neural changes rather than purely mechanical [11].

Current consensus remains that DLBP is caused by structural and biochemical changes within the IVD, commonly due to internal disc disruption (IDD) with associated nociceptive sensitisation and potential eventual nociplastic pain [13]. IDD describes discs which exhibit internal structural compromise including annular fissures, disc collapse and mechanical failure, and these compromised structures are potential contributors to the pain phenotype without overt herniation or external changes [9, 14, 15, 16, 17, 18, 19]. Pain attributable to other structures, including the zygapophyseal joints, sacroiliac joint, vertebral body, neural elements, and surrounding musculoligamentous structures, is considered non‐DLBP by definition [9]. Purists further consider pain from disc prolapse causing neural compression, despite arising in proximity to the IVD, should equally be considered non‐discogenic in origin. It is worth noting that conditions associated with segmental instability, such as spondylolisthesis, may produce concurrent discogenic pain through the same mechanisms of structural disruption, nociceptive sensitisation, and neurovascular ingrowth described above, independent of any coexisting neural compression or radiculopathy. In such cases, the disc itself may remain a legitimate pain generator, and DLBP should not be excluded solely on the basis of a coexisting structural diagnosis, provided the discogenic component can be reasonably isolated clinically.

The pathogenesis is often triggered by mechanical annulus injury creating microtrauma and endplate damage [18]. Acute or chronic loading injuries disrupt nutrient diffusion, initiating IVD metabolic dysfunction [20, 21]. Local cellular metabolic dysfunction is recognized as an integral component of the DLBP and IDD pathogenesis. It is characterized by increased catabolism, decreased glycolysis, mitochondrial dysfunction, and the production of reactive oxygen species, propagating a pro‐inflammatory and low pH environment, which further accelerates disc disruption [22, 23]. Other pro‐inflammatory states including Type 2 diabetes mellitus and obesity may further affect IVD function through adipokine‐mediated pathways [24]. Disruption may also begin with a reduction of nucleus pulposus cells with aging, reducing anabolic activity of the IVD [20]. The metabolic cascade may drive the pain phenotype of DLBP by promoting neovascularisation and aberrant nerve growth into previously aneural regions of the disc [15, 25]. Nociceptive sensitisation is therefore introduced into these newly innervated regions such as the inner annulus and nucleus pulposus. However, it is important to acknowledge the literature identifying a large degree of asymptomatic degeneration [26]. Furthermore, the cascade described by Kirkaldy–Willis shows that pain patterns evolve throughout life and may become less attributable to the disc itself, and more likely related to posterior element degeneration [27]. Within this definition, DLBP may encompass a spectrum of pain‐generating mechanisms. Nociception may theoretically be produced by normal physiological IVD loads creating abnormal IVD mechanical stress or strain secondary to structural changes, leading to depolarisation at normal mechanical thresholds. Alternatively, normal physiological IVD loads may also generate normal IVD mechanical stress or strain within proximity to abnormally sensitized nerves which may depolarise at lower mechanical thresholds. Both mechanisms may be further amplified by pathological neurovascular ingrowth into previously aneural disc regions. These processes may coexist in varying degrees within the DLBP population, and the heterogeneity of pain generation further compounds the diagnostic challenge.

Due to the inability to assess clinically the mechanisms above, the diagnosis of DLBP remains challenging and is one largely of exclusion [28]. Importantly, the diagnosis of DLBP encompasses two distinct components. The first is whether the patient suffers from DLBP at all, addressed through clinical history, examination, and investigation, potentially formalized through questionnaires and diagnostic algorithms. The second, and more challenging question, is which specific disc or discs are pain generating, which proves particularly difficult when multiple levels are degenerate, yet is critical for targeted treatment and surgical planning. As treatment options evolve, this will further develop into determining not only which disc is painful, but which treatment is most appropriate for each, demanding greater diagnostic ability. Additionally, although reasonable results have been described with operative management of degenerative disc disease, it is accepted that invasive treatment outcomes for patients with low back pain in the absence of clear diagnosis and clear operative pain generating targets can be inconsistent [29]. In the absence of a universally accepted diagnostic standard, clinicians must rely on clinical acumen, a constellation of nonspecific symptoms and imaging findings, and questionably reliable provocative discography findings [9, 30]. Additionally, clinicians lack accuracy in differentiating DLBP from disc prolapse pain on clinical assessment alone, given the overlapping symptoms and lack of known pathognomonic features [30]. Considering the burden of chronic low back pain and importance of accurate diagnosis for management, refinement of diagnostic features for DLBP is paramount. Optimizing outcomes in disc treatment ultimately depends on diagnostic precision, with evidence showing that interventions guided by a reliable reference standard yield significantly better results than those based on uncertain diagnosis [31, 32]. There are no existing reviews which summarize and synthesize the current diagnostic features of DLBP. This review aims to evaluate the diagnostic features, identify their limitations, and provide practical recommendations to improve future diagnostic research.

## 2 Methods

The study was conducted as a narrative review of the literature examining the diagnostic features of DLBP. This methodology was chosen to allow synthesis of heterogenous evidence spanning a variety of diagnostic feature domains. A search was conducted on PubMed, Scopus, and Web of Science with adjunct hand searching and citation tracking. Studies were included if they examined the diagnostic evaluation of DLBP, specifically focusing on clinical features or investigative modalities, both traditional and emerging. Key historical papers were included to provide essential context for the evolution of diagnostic approaches. No restrictions were placed on study design due to the exploratory nature of this review. Studies addressing overt neural compression, radiculopathy, or other non‐discogenic causes of low back pain were excluded to maintain diagnostic consistency. The selected evidence was thematically organized into major diagnostic domains to facilitate structured analysis and synthesis. Where available, diagnostic performance metrics were extracted from the most recent or highest‐quality studies. When multiple studies reported similar findings, greater weight was given to those with comparative reference standards or larger cohorts.

## 3 Clinical Features

The symptomatology of DLBP is not well established in the literature. While no historical or physical examination findings are pathognomonic for DLBP and many features overlap with other structural causes of back pain (vertebrae, zygapophyseal, and sacroiliac joints, neurovascular structures, and soft tissue), several features consistently appear in the literature as clinically suggestive and warrant consideration. The clinical features discussed in this section, and their respective diagnostic statistics where available, are summarized in Table 1.

**TABLE 1:** Summary of the clinical features of DLBP and their respective sensitivity (Sn), specificity (Sp), and positive likelihood ratio (LR+) where available from cited papers.

Clinical Feature	Sn (%)	Sp (%)	LR+
Aggravated by flexion in standing position [33]	52.4	93.3	7.8
Persistent pain between acute episodes [34]	—	—	4.1
Aggravated while washing face [33]	73.8	80	3.7
Centralisation [35]	—	—	2.8
Aggravated by prolonged sitting [33]	83.3	70	2.8
Mechanical vulnerability in the neutral zone [34]	—	—	2.5
Aggravated while standing after prolonged sitting [33]	83.3	63.3	2.3
Major loss of lumbar extension [34]	—	—	2.0
Aggravated with bony vibration [36]	71	63	1.9
Squirming in chair after prolonged sitting [33]	78.6	50	1.6
Passive sustained hip flexion	—	—	—
Aggravated by axial loading	—	—	—
Referred nondermatomal pain	—	—	—
Aggravated by sneezing/coughing/straining	—	—	—

### 3.1 Pain Pattern and Aggravators

Patients with DLBP experience years of chronic low back pain which persists between acute flare ups and has a poor natural history [34, 37]. The exacerbation of symptoms is consistently described as being provoked by activities which increase intradiscal pressure or involve sustained axial loading [9, 30, 38, 39]. Pain from DLBP is described as typically midline but may present paraxial, either unilaterally or bilaterally, with roughly equal distribution between these patterns [40]. The pain is commonly referred above the knee in a nondermatomal distribution, with higher‐level lumbar IVDs (L3‐4) often referring anteriorly, and lower IVDs (L4‐S1) postero‐laterally [41, 42, 43]. Occasionally referral below the knee is observed but this must be differentiated from radicular pain [13]. In line with the pathoanatomical mechanisms of pain generation, coughing, sneezing, and straining is reported to be a key feature of DLBP as this transiently increases intradiscal pressure [44]. Although classifying coughing, sneezing, and straining as aggravators for DLBP is supported by biomechanical plausibility, it lacks direct clinical data to validate the causation.

Flexion based movements are reported as aggravating factors due to the mechanical stress they place on the annulus [30, 33, 45]. However, it may be claimed that this symptom in isolation lacks clinical value due to its overlap with other pain sources. Among more specific aggravating factors, mid‐flexion aggravation such as during washing one's face while standing at the sink have demonstrated diagnostic value [33]. This may be explained by the increased mechanical stress placed upon the annulus fibrosus during flexion [44, 46], which, in the degenerate disc, is amplified as the nucleus pulposus loses proteoglycan content and hydrostatic pressure, which may offload greater stress to the annular fibers. In the context of aberrant nerve ingrowth extending beyond the outer third of the annulus, this increased annular stress may directly trigger nociceptive responses in newly innervated regions. However, the precise mechanism by which mechanical loading is transduced into nerve depolarisation and pain generation remains an area of investigation. Tonosu and colleagues provided valuable symptomatology data by administering a standardized medical interview to patients with MRI‐confirmed disc degeneration (Pfirrmann grade ≥ 4) in the absence of herniation or stenosis [33]. They compared responses between those who did and did not demonstrate ≥ 50% pain reduction following discoblock, as a surrogate for discogenic pain to establish sensitivity and specificity of reported aggravating features of their back pain. The authors reported the activity of washing one's face to have a sensitivity of 73.8% and specificity of 80%, yielding a positive likelihood ratio (LR+) of 3.69, proving a potentially useful clinical tool. Likewise, standing flexion demonstrated a LR+ of 7.82, proving flexion pattern to be a key characteristic of DLBP. Similar to standing flexion, other authors found functional limitations in sitting behaviors to be notable in patients with DLBP including aggravation after prolonged sitting or standing up after prolonged sitting yielding LR+ values of 2.78 and 2.27, respectively [30, 33, 47]. However, these authors did not quantify the duration of sitting defined as prolonged. Tonosu also reported ‘squirming’ (being unable to sit still and hold posture) in a chair as a novel finding in their survey, although this was a less predictive and more subjective finding (LR+ 1.57) [33]. One of the other diagnostically informative findings in their study was persistent pain between episodes of acute low back pain (LR+ 4.08), however, this is a very subjective finding that may struggle to translate clinically. The study conducted by Tonosu et al. provided valuable data on diagnostic clinical features, however, their control cohort was not based on response to discography, rather, assuming asymptomatic discs due to response from surgery for stenosis. Further, the medical interview used to gather their data was innately subjective, and responses may vary in their reliability.

Other mechanical features of interest, including moderate to major loss of lumbar extension (LR+ 2.01) and mechanical vulnerability in the neutral zone (semistooped or trunk twisted actions) (LR+ 2.5) have been reported; however, the low sensitivity reduces utility as screening tests [34]. Putos et al., however, suggest loss of flexion to be characteristic; however, this is based on a Delphi consensus model and has no diagnostic population data [30]. Additionally, confounding variables of other causes of postural instability may decrease the reliability of neutral zone vulnerability as a diagnostic feature. Overall, while flexion‐based aggravators may seem nonspecific, mid‐range flexion and prolonged sitting activities prove clinical value. The clinical utility of these features is likely to be maximized when interpreted in combination with other features and investigations.

### 3.2 Specific Movement Based Clinical Features

Centralisation is a phenomenon whereby referred discogenic pain may retreat toward midline in response to repeated movement testing, first described by McKenzie [48]. The original mechanical hypothesis proposed that repeated movement creates a shift in internal disc structures, redistributing nociceptive input toward more central regions. However, alternative neurophysiological explanations may be postulated, including repeated nerve depolarization leading to recruitment of adjoining neurones within the dorsal root ganglion. Localization of discogenic pain is inherently complex given the overlapping segmental innervation of lumbar structures, the diffuse cortical mapping of visceral pain, and the well‐documented referral patterns of lower lumbar nociception. Centralization is therefore best understood as a clinical phenomenon within a broader biopsychosocial pain framework, rather than a phenomenon explained by any single mechanism. Multiple strong studies have reported its clinical relevance and diagnostic accuracy with consensus that the high specificity but poor sensitivity may help differentiate discogenic from nondiscogenic back pain when positive [47, 49, 50]. However, centralisation relies on repeated movement testing for which some patients may not be able to complete; therefore, it is somewhat limited in utility. This was shown by Laslett et al. who demonstrated worse diagnostic utility of centralization in patients with severe disability when compared to minimal to moderate [49]. Current consensus studies and reviews suggest, however, that centralization phenomenon is an exclusive feature of DLBP [30, 51].

More recently, passive hip flexion has been described as a physical exam maneuver which may provoke patients with DLBP [52]. This technique was investigated as a predictor of LBP in the setting of provocative discography positive patients with IDD. DePalma describes this technique with the patient in supine; both straight legs are elevated passively by the examiner to approximately 45 degrees and then allowed to descend slowly. Similar to flexion aggravators, sustained hip flexion may be hypothesized to increase annular strain through a combination of axial compressive stress and circumferential tensile strain, with radial strain varying depending on the degenerative state of the disc, collectively provoking nociception in internally deranged discs. DePalma and colleagues demonstrated that pain reproduction during sustained hip flexion was significantly more prevalent in patients with IDD (95.8%) compared to those with facetogenic (55.8%) or sacroiliac joint pain (46.7%) [52]. Furthermore, the presence of midline pain during the maneuver increased the probability of IDD, while paramidline pain favored facetogenic or sacroiliac joint pathology, suggesting additional discriminatory value in differentiating pain sources. While promising, larger studies with robust diagnostic statistics referenced against a reliable reference standard are needed before routine clinical application.

### 3.3 Vibration Testing

The application of blunt electric vibrators to the spinous processes of vertebrae has been tested in provoking discogenic pain by creating mechanical stimulus for nociception. First introduced by Yrjama and Vanharanta as a fast, safe and effective test for DLBP, it proved a sensitivity and specificity of 71% and 63%, respectively [36]. However, the study included patients with radiculitis and has since been regarded as insignificant in distinguishing DLBP from other causes of chronic low back pain [53]. Despite this, additional research into its diagnostic utility may be justified.

### 3.4 Negative Predictive Features

It is equally important to recognize negative clinical findings that reduced the likelihood for DLBP. The presence of neurological deficits and radicular pain down a correlated dermatome should lower the index of suspicion as these are more suggestive of nerve root involvement [54]. Pain originating from the sacroiliac joint often presents with buttock pain that may or may not radiate down the leg [54]. A cluster of positive provocative maneuvers such as two or more positives in the Laslett sacroiliac joint battery may suggest sacroiliac origin, but diagnostic blocks are useful for confirmation [55]. The identification of facetogenic or vertebrogenic pain through clinical and imaging correlation should not be interpreted as exclusionary of concurrent DLBP, as multiple pain generators frequently coexist in the degenerative lumbar spine. Rather, these findings may reduce the index of clinical suspicion for DLBP as the sole pain generator [54]. Additionally, while a lack of pain relief with recumbency is not definitively diagnostic, it may reduce the clinical suspicion for DLBP as discogenic is generally accepted as a mechanical phenomenon [39]. It should be noted that while the presence of these negative features may reduce the clinical suspicion, it does not exclude DLBP but may prove it harder to clinically isolate.

## 4 Investigations

The mechanisms of DLBP make the nature of investigating it challenging. Structural disc degeneration on imaging is common in asymptomatic individuals and, in isolation, is insufficient for a diagnosis of DLBP [56]. While intradiscal metabolic changes are integral to the pathogenesis of both IDD and DLBP, the metabolic profile of a painful disc may differ from that of a structurally disrupted but asymptomatic one [31, 57, 58, 59, 60]. Quantifying these nociceptive metabolic markers represents a growing area of investigation, with the potential to discriminate painful from nonpainful discs rather than simply identifying degeneration. Currently, there is no available radiological or electrodiagnostic test which can be used to definitively identify the IVD as a source of low back pain [35]. The investigative modalities discussed in this section, and their respective diagnostic statistics where available, are summarized in Table 2.

**TABLE 2:** Summary of the investigations for DLBP and their respective sensitivity (Sn), specificity (Sp), and positive likelihood ratio (LR+) where available from cited papers.

Clinical Feature	Sn (%)	Sp (%)	LR+
Modic 1 Changes [61]	15	98	8.1
MRS [58]	100	80	5
Supine and standing X‐ray disc height discrepancy [62]	80	83.4	4.82
HIZ [63]	49	89	4.5
qCEST [64]	78	81	4.1
Ultrasound (with vibration testing) [65]	90	75	3.6
Discography [66]	81	64	2.3
Disc deformation on loaded MRI	—	—	—
Discoblock	—	—	—
Pfirrmann grade	—	—	—
DeVa	—	—	—
SPECT–CT	—	—	—

Abbreviations: DeVa = decay variance, HIZ = high‐intensity zones, MRI = magnetic resonance imaging, MRS = magnetic resonance spectroscopy, qCEST = quantitative chemical exchange saturation transfer, SPECT–CT = single photon emission computed tomography with computed tomography.

### 4.1 Magnetic Resonance Imaging

#### 4.1.1 Modic Changes

Vertebral endplate and adjacent bone marrow signal alterations seen on MRI are what Modic et al. first described in 1988 as being associated with disc dysfunction [67]. Modic changes represent the stages of vertebral endplate and subchondral bone response to disc degeneration and are classified into a three‐grade system: Type 1 indicates edema and inflammation, Type 2 reflects fatty infiltration, and Type 3 describes subchondral sclerosis. Among these, Type 1 changes have demonstrated the strongest association with DLBP when compared against significant pain on provocative discography, exhibiting a specificity of 98%, which supports a high positive likelihood ratio (8.10) [61]. Like many other studies, Thompson and colleagues used provocative discography as the reference standard, which carries its own inherent inaccuracies and may introduce circularity in the reported statistics. While the high specificity and positive likelihood ratio of Modic Type 1 changes indicate that their presence is strongly suggestive of DLBP, the very low sensitivity (15%) means that the absence of Modic changes carries little diagnostic weight, and the majority of painful discs will not exhibit these findings, limiting their utility as a standalone screening tool [61]. Importantly, Modic changes do not only correlate with disc disruption, but represent a spectrum of many pathological processes with potential transitions in phenotypes over time [68, 69]. Emerging evidence is also associating Modic changes with other forms of back pain including vertebrogenic [70]. Furthermore, Modic changes are not exclusive to symptomatic populations and population research reveals a substantial prevalence in asymptomatic individuals, highlighting the need for cautious interpretation [71]. Therefore, these changes are not pathognomonic, but rather suggestive of DLBP. When combined with other imaging or clinical findings of DLBP, Modic Type 1 changes may contribute meaningfully to diagnostic probability [61].

#### 4.1.2 Pfirrmann Grading

The Pfirrmann grading scale, widely recognized as the standard for MRI‐based assessment of lumbar disc degeneration, was first introduced in 2001 and has since been frequently utilized in relation to DLBP [72, 73]. It employs a five‐grade system based on disc structure, the distinction between the nucleus and annulus fibrosus, signal intensity, and disc height, with a higher grade indicating more severe degeneration [72]. A modified Pfirrmann grading system was described by Griffith et al. as an eight‐grade system to better describe disc degeneration in the elderly population [74]. While the Pfirrmann scale has demonstrated strong inter‐ and intrarater reliability, its sensitivity and specificity for DLBP remain debated [73]. Although degenerative changes identified by this grading are consistently observed in symptomatic individuals, similar findings are present in up to one‐third of asymptomatic patients [75, 76, 77]. Further, while higher Pfirrmann grades are more prevalent in symptomatic populations, the severity of degeneration does not correlate with pain intensity or functional impairment, and this correlation depends on the threshold used to define degeneration [78, 79]. Despite its wide mention in DLBP literature, there are no established diagnostic statistics published. Given the lack of strong association, the Pfirrmann grading scale is not a reliable tool to use when making a diagnosis of DLBP.

#### 4.1.3 High‐Intensity Zone (HIZ)

A HIZ is described as a focal hyperintense signal in the posterior annulus on T2 weighted MRI [80]. They have been extensively studied for their role in DLBP and shown to be correlated with discography findings [63, 81, 82, 83, 84, 85]. The pathogenesis of these HIZs is suggested to be related to annular tears in the outer third of the annulus fibrosus which result in inflammatory edema [86, 87]. Histological evidence shows granulation tissue and edema in discs with a HIZ which supports the pathogenesis and its role in identifying painful IVDs and annular disruption [86]. A recent meta‐analysis found that HIZ has specificity up to 89% but poor sensitivity of 49% for the diagnosis of DLBP when compared to discography, noting that these statistics are contingent on provocative discography as the reference standard, which itself carries well‐documented reliability limitations [63]. While controversial, chemical radiculitis may represent a false positive when interpreting HIZ, as leakage of nucleus pulposus can produce neuropathic leg pain from radicular involvement rather than true discogenic pain [88]. Therefore, while the presence of HIZ is strongly suggestive of DLBP, the absence does not rule out the diagnosis. Similarly, like Modic changes, though prevalent in symptomatic patients, HIZ can be found in asymptomatic patients and may not always be associated with pain on discography, limiting its use in isolation [81, 82]. While not definitive, HIZ provides structural evidence for potential DLBP and can be a strong finding used in context with other clinical findings.

### 4.2 Discography

Provocative discography is viewed by many as the controversial gold standard for DLBP. The technique first described in the 1940s quickly propagated that discs can be painful by injecting contrast medium and therefore flowing into radial tears and increasing intradiscal pressure [12, 89, 90]. These radial tears can be visualized on computed tomography (CT), and in 1987 the Dallas Discogram Description became a formalized tool used to describe the changes seen [91]. Osti and Fraser found discography to be more accurate than MRI for identifying symptomatic annular pathology [92]. While authors such as Bogduk have emphasized the value of this technique when strict operational criteria are used, Carragee and colleagues have highlighted its limitations, describing it as not highly predictive in identifying intradiscal lesions [9, 93]. The International Association for the Study of Pain (IASP) criteria describe the strict criteria echoing Bogduk's commentary: reproduction of the patient's typical pain of at least 7/10 during specific disc stimulation with < 50 psi injection pressure and the use of at least one control disc [94]. One systematic review of discography using the proposed criteria found sensitivity and specificity to be 81% and 64%, respectively, emphasizing its questionable use as a gold standard, although one meta‐analysis found a more robust false‐positive rate of 6% [66, 95]. Further, the complications of discography are well documented in the literature and warrant concern for its use. While rare, discitis is a serious complication with an incidence of 0.44% per patient and is difficult to treat due to the disc's avascular structure [96]. Data suggesting the use of styletted needles are suitable for prevention, where a larger needle is inserted through the skin to the annulus, and a smaller needle advanced into the disc [96]. Additionally, long term follow‐up studies have observed accelerated disc degeneration and increased incidence of new herniation in patients who have previously had discography performed [97, 98, 99]. However, when utilizing the IASP criteria, one study showed no significant increase in disc degeneration or herniation compared to controls [100]. Other rare complications include allergic response, nerve root damage, intrathecal hemorrhage, meningitis, acute IVD herniation, dural puncture headache, and vertebral osteonecrosis [101]. Potentially a large problem with the poor gold standard capability of provocative discography is widespread investigative inaccuracy. The reliance on an imperfect reference standard affects reported diagnostic accuracy for other modalities yet to be discussed.

Similarly, discoblock or analgesic discography has been described as a similar technique to provocative discography, where local anesthetic is injected into the suspicious disc instead of contrast medium [102, 103]. Confirmation of a symptomatic disc is done by substantial and immediate pain relief ≥ 50% following the injection and is often more acceptable to patients than provocative discography. Data show some patients with significantly better pain and disability outcomes at 3 years after using analgesic discography for selection for surgery compared to provocative discography [102]. However, it carries the same procedural risks as provocative discography [102]. There are no diagnostic statistics available for discoblock in the literature.

Molecular discography is described briefly as a technique which analyses IVD lavage fluid for metabolites. Fibronectin‐aggrecan complex and interferon‐gamma have been identified to be elevated in the DLBP population; however, these correlations are not strong and are also seen in negative control patients [60, 104]. This technique carries the same procedural risks as provocative discography.

### 4.3 Axially Loaded MRI

Among emerging biomarkers for DLBP diagnosis is assessment of disc deformation during loaded MRI [105]. A small novel study by Lagerstrand et al. compared this method with provocative discography as reference standard following the IASP protocol. In their small study, anterior disc height increase and posterior disc height decrease of the IVD were most predictive of pain provocation. While promising, results are underpowered and limited by assessing only the midsagittal plane.

### 4.4 Standing X‐Ray

Standing x‐ray is recognized as a limited adjunct in evaluating DLBP, by identifying IVD height loss indicative of degeneration [106]. Although, these findings are less specific for pain, and may be common in asymptomatic populations. However, one study found value in comparing supine to standing x‐rays [62]. The authors found a disc height discrepancy ratio of ≥ 6.04% between positions was able to identify patients with positive discography with sensitivity 80% and specificity 83.4%. While potentially valuable diagnostic data, the study was underpowered, and the results may be inflated.

### 4.5 Single Photon Emission Computed Tomography With Computed Tomography (SPECT–CT)

Adding to the standard CT imaging, SPECT–CT offers acquisition of a three‐dimensional data map, allowing precise localization and assessment of bone lesions and morphology [107]. SPECT images are created by detecting gamma rays emitted from radionuclides injected into the patient, which in this case would be Tc‐99 m methylene diphosphonate that binds to bone, specifically bony vertebral endplates. Through a process of coregistration, the radiographs from SPECT and CT are combined resulting in a three‐dimensional visualization of regions with increased radiotracer uptake [107]. Increased vertebral endplate uptake in SPECT–CT has been correlated with degenerative disc changes on CT and MRI, with strong associations to Modic Type 1 changes and Pfirrmann grades shown in a large cohort study [107, 108]. This is likely due to its strength in locating endplate changes, and because of this, its utility in differentiating discogenic from vertebrogenic back pain is unclear. Due to limited specificity in directly assessing soft tissue or disc pathology, SPECT–CT should not be used as a first‐line investigation, rather an adjunct when conventional imaging findings are inconclusive. Diagnostic specificity for DLBP remains incompletely validated, and attribution to the IVD is less certain compared to other sources of back pain.

### 4.6 Ultrasound

Ultrasound has been explored as a tool to assess lumbar disc morphology, particularly when combined with previously mentioned vibration provocation; however, it has not been widely investigated or adopted [65, 109, 110]. Ultrasound has been shown to be able to isolate IVD pathology; however, issues with technique standardization and surrounding soft tissue reduce reliability [65]. In isolation, ultrasound has poor diagnostic utility for DLBP, likely due to the technique being operator dependent; therefore, it is difficult to consistently visualize and classify structures as deep as the IVD [65]. When augmented by vibration provocation, it has sensitivity and specificity of 90% and 75%, respectively, for the diagnosis of internal annular fissures in the lumbar spine [65]. Despite this, the lack of literature and clinical uptake leaves ultrasound as experimental for DLBP diagnosis.

### 4.7 Newer Tools

#### 4.7.1 Magnetic Resonance Spectroscopy (MRS)

MRS operates on the same fundamental principles as traditional magnetic resonance imaging (MRI); instead of creating anatomical images, it can be used to detect and quantify metabolites in specific regions of the body [111]. The application of MRS to characterize metabolic markers associated with IVD degeneration was first demonstrated by Keshari et al., who employed ex vivo MRS methodologies [112]. In comparison with a population presenting with spinal deformities, patients with DLBP exhibited significantly lower ratios of lactate, collagen, and proteoglycan. More recently, Gornet et al. conducted the first MRS in vivo study of patients with DLBP, measuring data related to structural composition (collagen and proteoglycan) and tissue acidity (lactate, alanine, propionate) [31]. The study introduced and evaluated MRS‐SCOREs as diagnostic indices, reporting an initial sensitivity of 82% and specificity of 88%, with improved accuracy observed in nonherniated discs. In 2023, the same group further demonstrated the clinical utility of MRS by showing that surgical intervention guided by MRS‐based diagnoses yielded superior long‐term outcomes compared to those guided by provocative discography [57]. In this study, patients were stratified according to NOCISCORE thresholds derived from customized post‐processing of individual disc data. In 2024, Gornet et al. proposed the MRS as a new gold standard for DLBP, showing a sensitivity of 100% and specificity of 80%, further substantiating it as a diagnostic tool [58]. Although MRS is proving to be a promising advancement in the noninvasive diagnosis of DLBP, current studies have small sample sizes which may bias results. Additionally, post‐processing costs and availability may pose access limitations to socioeconomically disadvantaged populations. Despite this, the current work published by Gornet and colleagues provides compelling evidence for MRS as a reference standard for DLBP. When comparing published diagnostic statistics directly, MRS demonstrates superior sensitivity and specificity to provocative discography (Sn 100%, Sp 80% vs. Sn 81%, Sp 64%), yielding a markedly higher positive predictive value and LR+ (5.0 vs. 2.3). At minimum, MRS should be regarded as noninferior to provocative discography, with the available evidence suggesting superiority, pending larger prospective validation in independent cohorts.

#### 4.7.2 Quantitative Chemical Exchange Saturation Transfer (qCEST)

Adding to the growing biomarker assessment for DLBP, qCEST is an MRI measure of pH sensitive exchange rate of the glycosaminoglycans' and water protons in the nucleus pulposus [113]. Previously validated in porcine models [113], it has now been explored in small human populations [64]. Pelled et al. assessed a small cohort, finding that a ratio of 642 between qCEST and normalized T2 MRI signal yields sensitivity of 78% and specificity of 81% compared to provocative discography. While less predictive than MRS, it proves a valuable tool, having stronger LR+ and less risks than provocative discography. As mentioned, this study was small and did not include an asymptomatic control to get their statistics, potentially biasing results.

#### 4.7.3 Decay Variance (DeVa)

A novel MRI postprocessing technique pioneered by Sheldrick and colleagues named DeVa utilizes a T2* multi‐echo gradient echo sequence to assess microstructural IVD changes [59]. The resulting DeVa score reflects the concentrations of water and glycosaminoglycans, serving as a proxy for disc health [114]. DeVa scores have been validated against histological scoring of IVD degeneration in rabbits, signifying their reliability [115]. More recently, Sima et al. published the first human study of DeVa in 2025, revealing a strong association between DeVa scores and Pfirrmann grades [116]. Notably, DeVa demonstrated superior sensitivity in detecting subtle degenerative changes not readily identified on conventional imaging; however, the only studies currently are small and preliminary with no diagnostic statistics available on correlations with discogenic pain. While still in its early stages, DeVa shows promise as a non‐invasive tool for identifying degenerative IVD changes, regardless of symptomatology.

### 4.8 Clinical Implications and Future Directions

While no single clinical feature has sufficient diagnostic merit to confirm DLBP, their combination may raise clinical suspicion and guide further diagnostic workup. Further research is warranted to validate these features against a diagnostic standard. Moreover, the development of a pre‐test probability score incorporating these findings could significantly enhance diagnostic accuracy and provide practical value in clinical settings.

### 4.9 The Future of Clinical Practice: Diagnostic Algorithms and Clinical Prediction Models

Given the lack of a reliable reference standard for DLBP, combining multiple clinical and imaging features in a structured algorithm or prediction model may enhance pre‐test probability and diagnostic accuracy. While promising data is growing for individual diagnostic features, consensus lacks, and few studies observe these clinical features in combination. Laslett and colleagues assessed the diagnostic accuracy of clinical signs and self‐reports in combination with centralisation compared to provocative discography [34]. They found that combining features such as a history of persistent low back pain between acute episodes, a sense of vulnerability when semistooped or twisting, loss of lumbar extension, and centralization yielded high specificity (95%) but low sensitivity (30%), limiting its use as a screening tool. Additionally, the features of visualized extension loss and feeling of vulnerability are innately subjective in nature and lack reliability. Similarly, Tonosu et al. studied their five‐question medical interview for the most diagnostic tool for diagnosing DLBP [33]. Items included pain after sitting too long, while standing after sitting too long, squirming in a chair after sitting too long, pain while washing one's face, and pain in standing position with flexion. When four of the five items were positive, the tool demonstrated a sensitivity of 100% and specificity of 71.4% compared with discoblock outcomes, suggesting good potential as a non‐invasive screening aid. However, external validation and assessment across diverse populations are still needed before routine clinical application.

An algorithm proposed by Lorio et al. in 2025 developed a decision tree using key features of DLBP informed by a Delphi consensus from an 11 expert panel [117]. While the proposed algorithm represents a promising step toward a standardized algorithm, it has yet to be validated in clinical populations and includes some questionable steps. Notably, the decision tree centralizes Pfirrmann grading severity in the radiological evaluation step, an approach which may have questionable validity given the lack of specificity noted in literature [75, 76, 77]. From the findings in this review, perhaps other more reliable investigative methods such as MRS could have been included to enhance its validity. MRS is referenced only as an optional consideration for patients without structural MRI changes, representing a significant underutilization of the available evidence. MRS may offer an important keystone in the development of a prediction model. Unlike conventional MRI, which lacks specificity, MRS provides metabolic markers that strongly correlate with painful discs [31, 57, 58]. Although early, current available diagnostic statistics are at least equivalent to and, by published metrics, superior to those of provocative discography, which, if incorporated into a diagnostic tool, may substantially strengthen its validity while avoiding the invasive risks associated with discography. However, MRS focused on lactates may be an issue in themselves as lactates are potential energy sources for the disc, which has been demonstrated to increase disc height in exercising rat experiments [118, 119].

**FIGURE 1:**
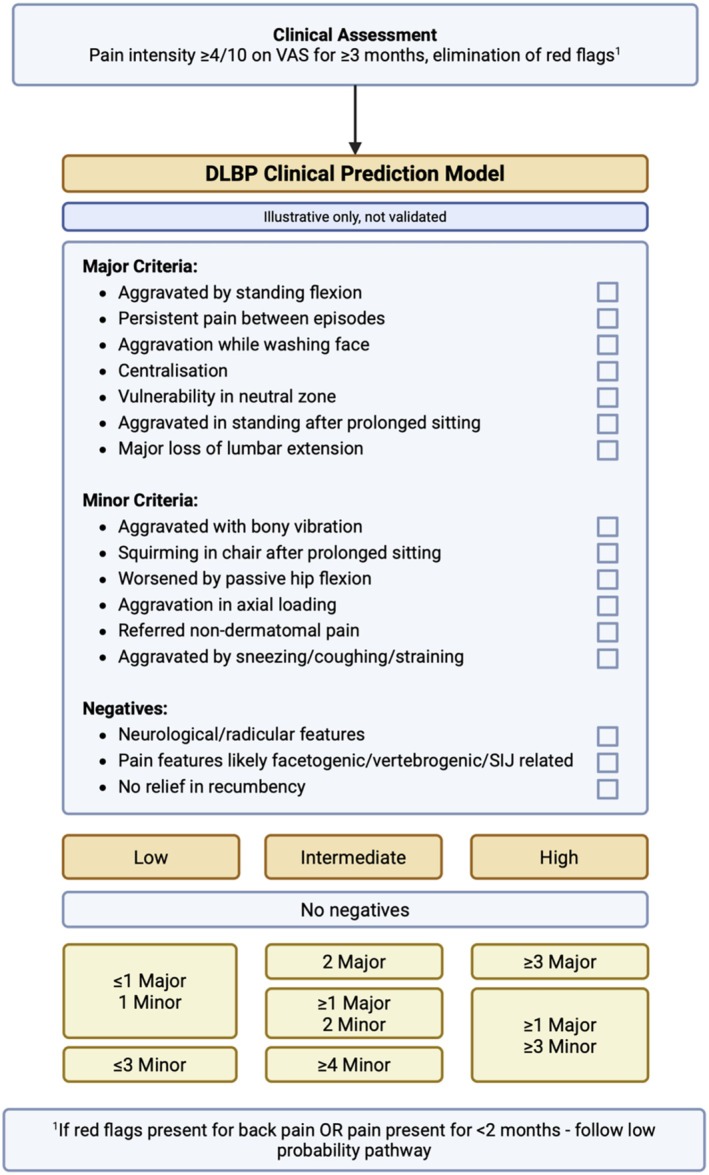
Proposed DLBP clinical prediction model.

An illustrative clinical prediction model is shown in Figure 1, which was developed using the synthesis of features reported in this review. Given the poor specificity imaging evidence of degenerative discs alone in relation to DLBP, the model prioritizes the clinical phenotype associated with DLBP. Rather than pursuing a radiological standard, patient signs and symptoms are used to guide the selective use of imaging. This approach recognizes that disc degeneration is common and frequently asymptomatic, and the diagnosis of DLBP requires concordance between the clinical phenotype and supportive imaging findings. The proposed major and minor criteria were determined with respect to their LR+, with major criteria requiring LR+ ≥ 2. Restricting assessment to patients with persistent and significant low back pain of greater than 3 months duration (subacute) is, particularly, relevant given the favorable natural history of low back pain in the majority. The DLBP pathogenesis is one of chronic sensitization, whereby acute presentations are less likely to be discogenic in origin [2, 9]. By risk stratifying patients into “low, intermediate, and high” probability groups, care pathways and recommendations may be tailored. Patients with low probability should be reassessed for other differential diagnoses. Intermediate and high probability patients may be assessed further with supplementary imaging (MRI, CT, SPECT) with consideration of the new novel techniques (MRS, DeVa, qCEST). If patients are surgical candidates, provocative discography may be considered to confirm symptomatic level if unclear. While not validated, this framework is broadly based on commonly accepted and reported clinical features of DLBP, but must be subject to formal diagnostic assessment before use. This tool provides a conceptual basis for the development of future prediction models, including the possibility of machine learning and artificial intelligence input. The implementation of such a model in clinical practice may have capacity to improve imaging and healthcare resource stewardship. By reducing overimaging, it may reduce patient harm from nocebo effects of incidental findings, lower healthcare costs, and lessen the environmental burden associated with advanced imaging modalities [2, 120].

Future studies should aim to develop and assess clinical prediction models which serve as adjuncts in diagnosing DLBP clinically. The scoring may be based on a cumulative score or a weighted model, where individual clinical features are assigned values according to their relative diagnostic strength using positive likelihood ratios. Clinical predictors should be collected using a synthesis of the literature with the consideration of additional expert consensus. Not included in this review, but of noteworthy mention, flexion relaxation ratio should be studied in the DLBP population and included as a potential clinical biomarker. Current literature only investigates non‐specific chronic low back pain populations and shows it as a reliable and reproducible biomarker which can discriminate between healthy and chronic nonspecific low back pain populations [121, 122]. DLBP is chronic, and with similar neuromuscular changes potentially occurring, investigating flexion relaxation ratio as a specific biomarker may be of value in this population. Additionally, future studies may assess the presentation of pain in DLBP as being nociceptive or neuropathic using the painDETECT tool [123]. Differentiating between pain presentations may provide clarity as association between DLBP and imaging features evolve. Clinical predictors in the model should be validated preferentially against MRS as the emerging non‐invasive reference standard of choice, or against provocative discography where MRS is unavailable. Using cut‐off scores, the diagnostic utility may be assessed accordingly, with MRS offering the advantage of avoiding the invasive risks and reliability concerns inherent to discography. Such models should undergo both internal and external validation in independent cohorts to ensure generalisability. Beyond statistical performance metrics, assessment should also consider clinical applicability, feasibility in routine practice, and the potential to reduce reliance on invasive and often costly diagnostic procedures. This review provides a foundation for the development of future diagnostic models that integrate clinical features with investigative findings.

## 5 Conclusion

This review has summarized the current literature on diagnostic features of DLBP, highlighting the ongoing challenges in accurate identification, and proposing a conceptual framework for a clinical prediction model. At present, no clinical features have been consistently validated as predictive for DLBP. While activities involving flexion and prolonged sitting demonstrate some predictive value, traditional hallmarks such as axial loading pain and symptom aggravation during coughing or sneezing remain theoretical and unsupported by robust evidence. Evidence of clinical features demonstrated in large diverse cohorts is lacking. Discography is limited by concerns over reliability and safety, whereas conventional imaging techniques often lack specificity. Other techniques including supine to standing x‐ray, ultrasound with vibration testing, and disc deformation lack sufficiently powered data. Emerging modalities, such as MRS, qCEST, and DeVa, show promising early potential in detecting metabolic markers that may serve as objective diagnostic indicators. MRS warrants consideration as a reference standard given published diagnostic statistics that are at least equivalent, and potentially superior to provocative discography, without the associated invasive risks. Despite the growing body of published features, clinicians continue to face difficulty in reliably identifying DLBP. Importantly, the presence and severity of disc degeneration on imaging alone do not correlate with pain, reinforcing the need for diagnostic frameworks which prioritize clinical signs and symptoms. The proposed illustrative clinical prediction model provides a conceptual framework for future research and development, with potential to guide more accurate diagnosis and reduce unnecessary imaging.

## Author Contributions

**Ashish D. Diwan:** conceptualization, investigation, writing – review and editing, methodology, supervision. **Mario Giuseppe Zotti:** conceptualization, investigation, writing – review and editing, methodology, supervision. **William Roger Peters:** conceptualization, investigation, writing – original draft, methodology, writing – review and editing, data curation.

## Funding

The authors have nothing to report.

## Ethics Statement

The authors have nothing to report.

## Consent

The authors have nothing to report.

## Data Availability

The data that support the findings of this study are available from the corresponding author upon reasonable request.
